# An Economic Analysis of Brownfield and Greenfield Industrial Parks Investment Projects: A Case Study of Eastern Slovakia

**DOI:** 10.3390/ijerph18073472

**Published:** 2021-03-27

**Authors:** Henrieta Pavolová, Tomáš Bakalár, Alexander Tokarčík, Ľubica Kozáková, Tomáš Pastyrčák

**Affiliations:** 1 Institute of Earth Resources, Faculty of Mining, Ecology, Process Control and Geotechnologies, Technical University of Košice, Letná 9, 042 00 Košice, Slovakia; Henrieta.Pavolova@tuke.sk (H.P.); Lubica.Kozakova@tuke.sk (L.K.); Tomas.Pastyrcak@tuke.sk (T.P.); 2 Energy Cluster of Prešov Region, Levočská 12, 080 01 Prešov, Slovakia; klasterekpk@gmail.com

**Keywords:** investment project, industrial park, simple additive weighting, brownfield, greenfield

## Abstract

The implementation of industrial park investment projects in relation to the use of brownfields and greenfields is a constantly debated issue. Brownfields are unused areas, often with devastated building objects and environmental burden that pose potential risks to the investor but also contain the possibility of using the available infrastructure and facilities for the use of the proposed investment project. The objective of the study was to assess the positive and negative investment of these types of sites based on the available information on the possibilities of investing in the implementation of the brownfield industrial park compared to the greenfield in the western part of East Slovak region and to identify a more appropriate alternative of investing. Based on the assessment of investment in the industrial parks, the appropriateness of the allocation of investment capital was assessed through the simple additive weighting (SAW) method. The SAW approach allows us to objectivize the weighting value of selected factors and thus assess the appropriateness of the allocation of investment capital. Based on the results, it is more advantageous to allocate the investment capital to the greenfield as the return on investment of the project expressed as a percentage of the average annual profit was 9.5%, compared to brownfield with only 2.9%.

## 1. Introduction

Nowadays, the addressing the problem of brownfield and greenfield potential in the development processes has been oriented toward the principles of sustainable development. Slovak regions are characterized by interregional disparities with a significant difference in economic attractiveness and increasing polarization of the Bratislava region compared to other Slovak regions. Brownfields and greenfields must be understood as a key factor of progress, modernization, and adaptability in the newly created conditions determining the development of society in all areas of the regional structure. Brownfields, regardless of their previous use and cause of creation, represent a hidden reserve for the recovery of the Slovak economy and an increase of the competitiveness of the regions. Brownfields and greenfields, in the processes of regional development, are necessary for public interventions through formal and informal tools. The issue of regional development defined in this way requires the elaboration of an independent and comprehensive strategy at the regional level based on precisely defined conditions and other requirements through legislative measures [1,2,3].

Sustainable land management includes the re-use of brownfields, as greenfields have become scarce in densely populated areas [4,5]. The prioritization of potential brownfield sites requires a large amount of data such as site-specific data, and data on engineering, financing, and policy aspects to differentiate the sites in making the selections [6,7,8]. In Southeast Europe, there is a trend of greenfield investment, and the awareness of investing in brownfields is gradually rising [9]. The basic failures of brownfields re-use are the lack of adequate vertical—at the levels of the state, region, and municipality—and horizontal coordination—among the public institutions, private investors, local governments, and citizens [9,10], as also confirmed by Pakistan studies [11,12], an Indian study [13], and a Slovak study [14]. Soft re-use of brownfields, that is changing into greenfields, for example for civic amenities [15], crops [16], or other uses [12] where the soil is untouched and not polluted in any way, can provide valuable services both in urban and more rural contexts, but their monetization might be uneasy [17]. However, it must also be mentioned that tools and support for land management decisions are limited. Each investment project is different due to the size and scale that affect the process and often set the tools for decision-making processes, as there are many aspects that must be considered [4]. In addition, there are no adequate strategies and experts for solving the problem of brownfields [18]. Other economic risks may be unclear property rights and lack of information on the kind and extent of contamination [19]. Despite all the negative aspects, the multiple environmental, economic, and social benefits of brownfields and greenfields should be considered a sustainable land-use policy [11]. A case study from Hong Kong [20], a city that has similar local conditions—culture, transport, and land use policies—in built-up and green areas, revealed that the establishment of transit infrastructure precedes urban development, any built environment can be easily reshaped, and new planning concepts can be introduced in greenfields. From a sustainable land-use perspective, although it cannot be taken as a rule, brownfields regeneration is prioritized over greenfields development [21]. Another option of “greening” brownfields and greenfields is community gardens, which might change the socio-economic characteristics of a neighborhood by increasing incomes, changing the current residents and businesses by wealthier people moving in, improving housing, and attracting new businesses (gentrification) [22]. So-called “green gentrification” is connected to green spaces that are built in remediated and redeveloped commercial and residential buildings [23,24].

The primary aim of the study was to assess the positive and negative aspects of investment into brownfields and greenfields. The analysis was based on the available information on the possibilities of investing into a brownfield industrial park compared to the greenfield in the East Slovak region to identify a more appropriate alternative of investing. Fulfilling the defined aim, it was necessary to create a comprehensive overview of brownfields and greenfields investment projects for the implementation in industrial parks and, by globalizing the available information, to point to their strengths and weaknesses as well as the opportunities and threats that could determine the decision on placing the investment capital. Based on the detailed analysis of selected indicators of the brownfield and greenfield investment project in the East Slovak region, the more appropriate alternative for the allocation of investment capital was identified.

**Figure 1:**
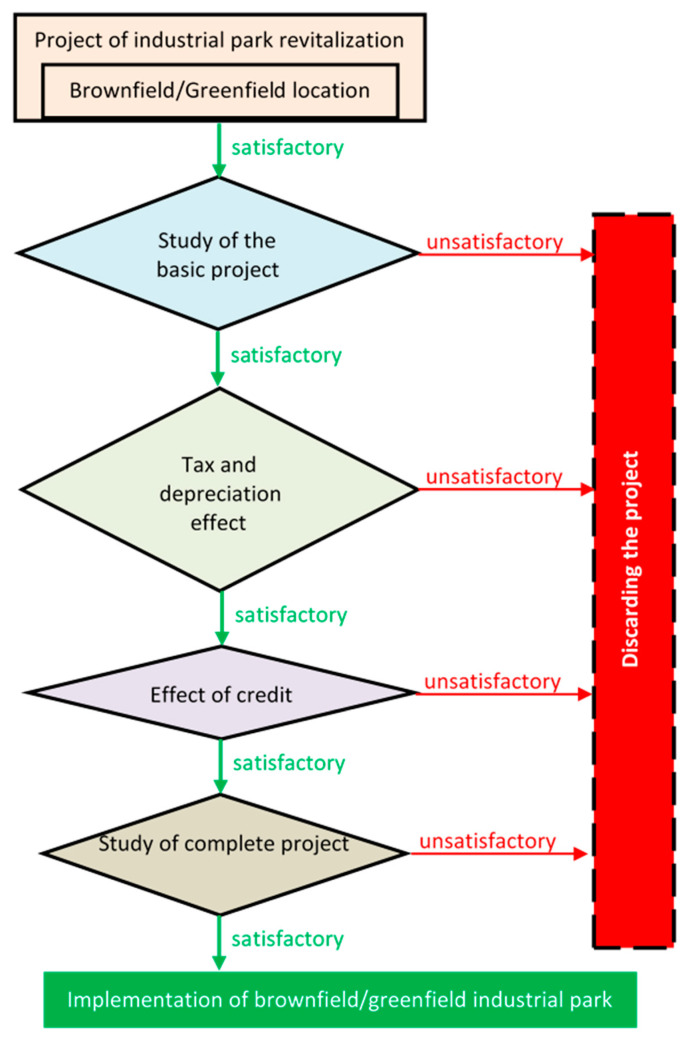
An assessment of the economic efficiency of an investment.

Potential investors, when deciding on strategic investments in industrial parks and their location, are strictly controlled by very detailed economic analyses based on a detailed study of the economic efficiency of the investment [25,26] (Figure 1). The detailed study verifies all achievable engineering, environmental, legal, and economic information, including environmental impact assessments, which, in the case of brownfields, are a decisive factor affecting the investor’s investment.

When evaluating a project, the most important fact is whether the economic development of the project guarantees positive results and economic returns on the investments made [27]. The actual expression of the measure is not essentially important because the real environment that the project will be in will be considered at the following levels. The knowledge of how to set up and equip brownfields, as well as to compile appropriate revitalization brownfield models, is a prerequisite for both searching successful brownfield sites and evaluating their development potential for industrial park areas [28,29]. For industrial investors who are coming to the domestic market, there are very attractive corporate tax deductions that they can apply for in several consecutive tax periods, or they have so-called “tax holidays” [30]. Other benefits that investors use are support from the Office of Labor, Social Affairs, and Family to create jobs, especially for disadvantaged job seekers [31,32,33].

The above-mentioned incentives lead to an extensive construction of industrial parks as well as other commercial complexes in individual regions, and these trends must be perceived and assessed from the point of view of three interrelated sustainable development pillars—economic, socio-cultural, and environmental [31,32,33,34].

## 2. Background

Brownfields are the result of changes in the functional use of the area and represent a huge environmental problem and a barrier to spatial development of municipalities. If brownfields are in an attractive area, their environmental burden is only mild; potential investors or new owners can invest in the revitalization of such territory and re-use it to profit [35]. Considering severely damaged areas, the normal rules of demand and supply cannot guarantee their recovery. The cost of recovering them is higher than the value of the land as discussed above. Finding new investors for such areas is challenging, because it is better for a potential investor to buy an undamaged land: greenfield. The approach to solving brownfields differs according to their character and economic aspects [36,37], which are described in Table 1.

**Figure 2:**
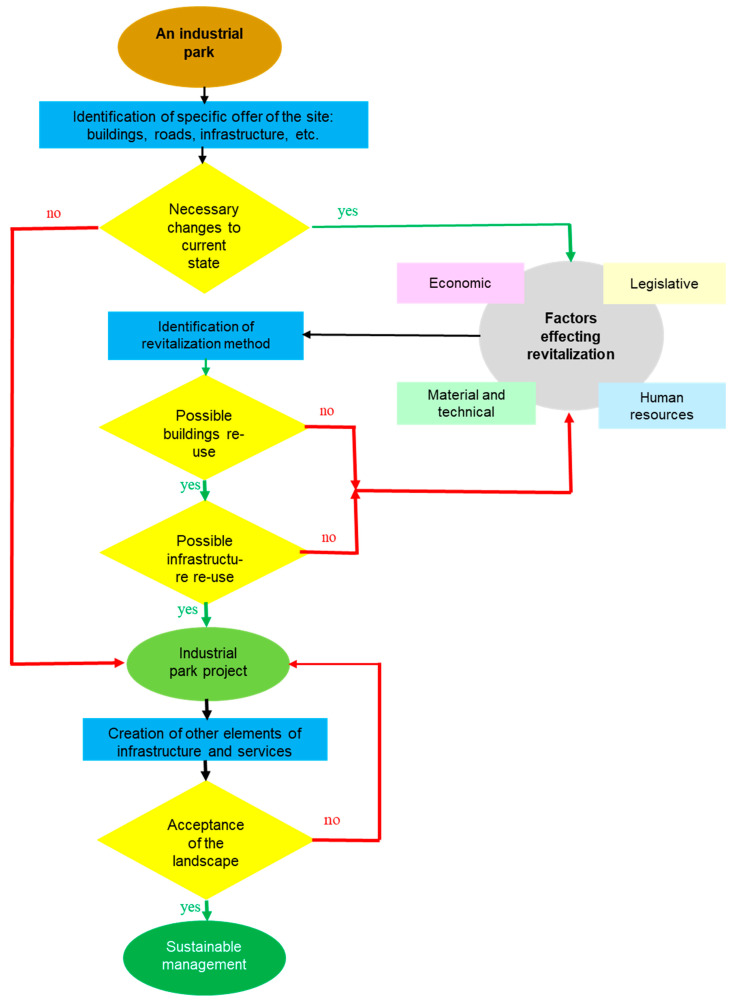
Model of industrial park investment projects management.

The revitalization of unused or economically inefficiently used areas and objects, i.e., brownfields, requires a systematic approach throughout the development of the state, regions, urban agglomerations, and individual municipalities [28]. Identifying and analyzing the environmental burden of contaminated sites, redeveloping them as well as re-utilizing and revitalizing brownfields represent considerable financial costs, especially for public as well as private budgets. However, in the long term, this is a very effective process that is fully in line with the principles of sustainable development of towns and municipalities [38]. It is for these reasons that finding solutions for brownfields is an important part of an effective spatial planning policy that focuses on the urban territory and especially the cities. Such cooperation should identify the whole range of complex challenges posed by the revitalization of these sites in industrialized countries and explore ways of sustainable management of brownfield sites (Figure 2) [36,37], the spatial plan itself being described as a sophisticated model of broader relations which has the following characteristics [39]:

- Shows how individual buildings and open spaces will be linked.
- Defines the height, overall geometry, and size of buildings, describes proposed relations between buildings and public spaces.
- Determines the distribution of the provided activities or use.
- Identifies the system of movement of people—walking, cycling, car or public transport, service, and vehicles for waste disposal.
- Establishes the basis for the provision of other infrastructure elements and public services.
- Combines the physical form with the socio-economic and cultural context and the interests of all concerned.
- Allows understanding how well an industrial park is integrated with the surrounding context of the landscape and the natural environment.

## 3. Objects of Study and Methods

Starting from the precisely defined objectives, several methodological procedures and methods of exploring and interpreting results, for example, in dealing with relatively complex problems of implementation of brownfield and greenfield investment projects, including methods of analysis, synthesis, deduction, comparison, simple observation, as well as simple additive weighting (SAW, or so called “proportionally-index analysis”) by the weighted sum were used.

The study industrial park for the investment project is in the southwestern part of the East Slovak region, which is located in a brownfield area of 80,000 m^2^ with a built-up area of about 28,322 m^2^, and it is an environmental burden due to previous mining activities. The industrial park is designed as a functional area of the industrial production of former mining activity. There are no major objects or water resources in the territory, and the territory is not a protected natural landscape area. The investment project of the industrial park is affected by the protective zone of the railway of the Slovak Republic, but its character is not in contradiction with the conditions of implementation in this zone.

**Figure 3:**
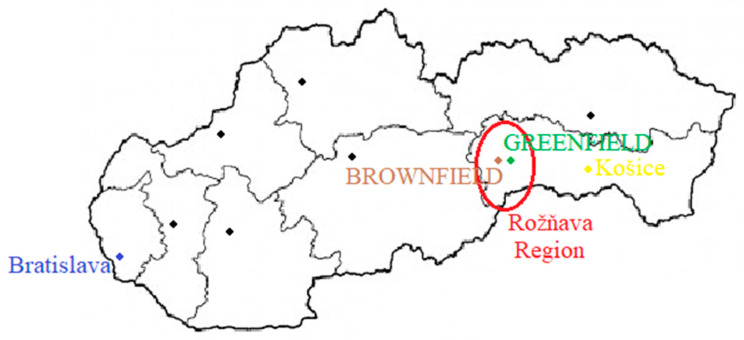
An indicative map of the location of brownfield and greenfield investment projects.

The study green area for the investment project is in the same area of the southwestern part of the East Slovak region with an area of 80,000 m^2^ and no built-up area. There is no environmental burden (Figure 3).

For the purposes of identifying the most appropriate investment capital allocation strategy for brownfields compared to greenfields, the SAW analysis [40] was conducted. Afterwards, the brownfield and greenfield investment projects in the southwestern part of the East Slovak region were compared.

The necessary relevant data on the status of brownfield regeneration and the development trends of the individual regions of the Slovak Republic were obtained mainly from professional publications and manuals of Slovak and foreign authors, legislative regulations, and various EU statistical databases, etc.

Several methods evaluating and interpreting the results of the assessment of investment projects of industrial parks on brownfield compared to greenfield were used. Thus, the problem is based on the systematic and quantitative analysis of predefined indicators. Based on the assessment of investment in the industrial parks, the appropriateness of the allocation of investment capital was assessed through the SAW method [40] using Equation (1):

*U*_m_(*x*) = Σ*α*_i_.*u*_i_(*x*_i_) (1)

*U*_m_(*x*)—overall usefulness of the evaluated project (stability index), *m* = 1, 2, 3, …, *m*,
*α*_i_—the weight of the *i*-th criterion defined by the decision-making body; Σ*α*_i_ = 1,
*u*_i_(*x*_i_)—the usefulness of the *i*-th criterion for *x*_i_, where often *u*_i_(*x*_i_) = *x*_i_,
*x*_i_—the value of the result according to the *i*-th criterion.

A certain weight was assigned to each predefined factor *α*_i_ accepting the generally valid condition of Σ*α*_i_ = 1. The weighing value was objectivized; that, is the individual factors, from which the matrix was created, were compared, working with values of 1, 0.5, and 0. On the diagonal of the created matrix, the value of 0 was plotted. If the factor considered was more important than the one it was compared to, the value of 1 was plotted; if it was less important, the value of 0 was plotted, and if it was of the same importance, the value of 0.5 was plotted. The rows were summed to reflect the mutual interactions of factors, which quantified the final value of the weight $\alpha_{i}$ of the factors. These factors were assigned points 1–5. The more points attributed to the rated factor, the greater the likelihood that this factor is fulfilled. The points were multiplied by weights, and their amount determined the resulting number of points for an efficient industrial park implementation project [40]. When processing the data, the basic methods of descriptive statistics and the basic statistical methods were used.

The SAW method [41,42] is a multi-criteria decision-making method. The decisive body determines the input parameters and their dependencies. It is a proportionally index method that can lead to specific choice by comparing overall evaluations [43,44,45]. It applies all the criterion values of an alternative and makes use of regular arithmetical operations—addition and multiplication [46,47,48]. The properties of SAW, including conditionally monotonic with utility and risk neutrality of the decision behavior, are essential for determination of the weights of each criterion [49]. In dealing with both qualitative and quantitative dimensions, the SAW method was also merged with other fuzzy analyses [50] to answer fuzzy multi-criteria decision-making problems and include uncertainty and vagueness [51]. The SAW method was used in a comparison of coal power plants with nuclear power plants with a result stating that the coal power plants with carbon capture and storage are slightly more efficient than nuclear power plants [52]. The SAW method was used in a study of the impact of anthropogenic risks on protected areas [53], in the selection of obtaining methods for small building works [54], in risk assessment and evaluation of pollution by metals in a copper sulfide mine in Iran [55], in the prioritization of criteria and indicators for the evaluation of environmental, economic, and social factors in the sustainability of national parks [56], in evaluation of ecological and human-related parameters affecting industrial zoning [57], and in the assessment of risk management capability based on accepted risk allocation principles, which were derived from qualitative data and expert knowledge [58]. Multi-criteria decision-making methods—an order preference by similarity to ideal solution – Technique for Order of Preference by Similarity to Ideal Solution (TOPSIS) and SAW methods—were applied for the identification, customization, weighting, prioritization of criteria, and indicators of evaluation for the determination of potential of environmental, economic, and social services [56].

## 4. Results

The economic benefit is the influx of foreign capital, partial contracts for some domestic suppliers, and competitive products. At the same time, there are disadvantages for existing domestic entities, especially small and medium-sized enterprises. Under no circumstances can they compete with economically strong multinational companies, using, in addition, a large-scale government investment aid scheme. In many cases, investors do not pay full financial costs, increasing the pressure to increase public budget expenditures.

### 4.1. Analysis

In the case of environmental aspects, the negative consequences clearly prevail. In most cases, investors decide to invest in greenfield investments when deciding where to locate their investment. Investors clearly prefer road transport and require new industrial agglomerations in a “free country” to be linked to capacity communications. Industrial and commercial complexes, as well as the newly built service infrastructure, are very often built on good-quality land with superior production capacity. They severely disturb the suburban cultural landscape and cause irreversible destruction of local ecosystems and long-term deterioration of natural resources in the built-up area and its surroundings (soil, water, biota) [59].

For the investor, investments on greenfields are advantageous, relatively fast, and, in the case of interconnected and generally prepared new industrial zones, also very cheap because a significant part of the costs are borne by the concerned municipality. Compared to greenfields, potential investors entering brownfields may calculate a much lower range of benefits, since there is no comprehensive state support program for investment in long-term unused and devastated areas or objects in the Slovak Republic. Despite this unfavorable fact, it is undisputed that as part of the revitalization of brownfields in a compact site, investors can use industrial parks for the following [60]:

- Existing transport and technical infrastructure.
- Existing manufacturing and other objects.
- Good connection to the regional and global freight transport system.
- Good connection to a functioning public transport system.
- Contact with existing subcontractors and services.
- Sufficient workforce in residential areas in the vicinity or within public transport.

It is clear to municipalities that the removal of old environmental burdens on their territory is accompanied by the subsequent improvement of the environment, the creation of new jobs, the re-development of business activities, the improvement of the affected locality, and the overall attractiveness of the site.

From the available data, individual investment projects ranged according to selected indicators, including:

- Investor density.
- Job density.
- Non-repayable financial contribution (NFC) per job.
- Occupancy of the industrial park.

All investment projects were categorized according to the achievement of the chosen indicator into three categories:

- A: Industrial parks achieving above-average results.
- B: Industrial parks achieving average results.
- C: Industrial parks achieving sub-average results.

For the investor density data, the A category of industrial parks consisted of greenfields (100%), the B category included brownfields and greenfields in the ratio of 80%: 20%, and the C category included brownfields and greenfields in the ratio of 20%: 80%.

Based on the available job density data, category B was only brownfields, while category A (1 brownfield and 2 greenfields) and category C (4 brownfields and 8 greenfields) had both. The NFC per newly created job and the occupancy of industrial park in categories A and B were 50% brownfields and 50% greenfields, while category C was the category where the highest NFC per newly created job was mostly greenfields—83%, and brownfields represented only 17% of industrial parks in this category.

From the results of the analyses of the implementation of subsidized brownfield and greenfield industrial investment projects from the Sectoral Operational Program—Industry and Services, brownfield investment projects with engineering networks and buildings that constituted the primary potential of their use were more effective. For this reason, the SAW method of decision-making for the purpose of defining an effective strategy for the allocation of investment capital to the implementation of industrial parks was implemented. On the basis of the results of the partial analysis in support of the implementation of industrial parks in interaction with investment capital location carried out so far, the necessary decision factors, including existing transport infrastructure (f1), existing technical infrastructure (f2), existing production facilities and other objects (f4), land acquisition cost (f4), good connection to the regional and global cargo transport system (f5), landscaping costs (f6), contact with existing subcontractors and services (f7), environmental burden (f8), additional costs of the investment project (f9), running costs (f10), and time schedule of the land preparation (f11), were identified. Identifying the factors of decision-making on the allocation of investment projects of industrial parks implementation, the quantification of weights *α*_i_ according to the methodological procedure was made with determination of their priority in the decision-making process and compilation in descending order (Table 2).

Based on the results of the comparison of the brownfield and greenfield industrial park investment projects using the weighted sum (Table 3), the implementation showed greater benefits in investment capital allocation for brownfield industrial parks.

### 4.2. Results of the Analysis

The industrial park investment project is in the southwestern part of the eastern Slovak region, as described above, which is affected by the industrial conversion of mining and related production activities by not only the loss of the economic base, the increase in registered unemployment, but also extensive devastated mining areas threatened by environmental burden. The brownfield industrial park investment project must be in compliance with the indicators determining regional development, namely the following:

- Arranging the development of business in industry, reducing registered unemployment, and improving the life quality of the population.
- Implementation of interactions between public administration, investors, entrepreneurs, and subcontractors, which determine the competitiveness of the industry in the region.
- Reclamation and modernization of former industrial facilities after mining activities for the sustainability of investments.

Prior to commencing work, it is necessary to identify all engineering networks within the building site and to prepare the area where the construction sites are devastated after mining. At the same time, it is also necessary to cut the trees and bush in interaction with securing green planting. At the brownfield site, there are buildings that need to be demolished to provide the necessary space for the strategic investor, as some objects are severely devastated due to weather effects and long-term non-use. Upon closer examination of the industrial area, there are seven unusable objects. The rough terrain work follows the demolition work of the buildings. On the territory of the future industrial park, an engineering–geological survey was carried out, pointing out that the earthworks will be carried out mainly in soil of clay with gravel with low to medium plasticity. Due to the composition of the terrain, it is necessary to do rough terrain modifications in a part of the area. Coarse landscaping is specified by expert estimation. Expected earthworks within the rough terrain work include demolition, excavation, trench of trees and shrubs, and landscaping with compaction.

The entire area of the industrial park area is not expected to have a negative impact on the mining areas, since the mining passages are outside the brownfield and at depths above 100 m below the surface. Due to the complexity of the terrain regarding its composition, the extent of the leveling, the altitudinal orientation, and the presence of the remains after the previous construction activity (various building pits, foundations, masonry, and construction rubble after the demolition of the original buildings), the residuals must be processed based on expert estimation. This expert estimate will need to be corrected and adjusted directly during construction work, which will be followed by building supervision for the investor. Cubic capacities will be consulted during the works at the investor–supplier level.

## 5. Discussion

The issue of using brownfields and greenfields for industrial park investment projects is a constantly discussed topic at the local, regional, and supra-regional level of the European Union. Brownfields are unused areas, often with devastated building objects and environmental burden, which pose potential risks to the potential investor, but they also present the possibility of using the available infrastructure and facilities for the investment project. Indeed, the implementation of brownfield industrial park investment projects also supports the objectives of regional development within the EU in the social, economic, and environmental areas, and it should be explicitly supported by investment interventions from the state, particularly in the private sector. It is precisely these facts that should persuade potential investors to implement brownfield industrial park investment projects in the sense of long-term sustainability of a particular investment project in a local, regional, or supra-regional market.

Economic, social, and environmental benefits and costs associated with a brownfield and greenfield redevelopment in the Greater Toronto Area, Canada were compared, while brownfield redevelopment resulted in benefits to citizens and the main benefit was avoiding high transportation costs for people living in the peripheral greenfield areas and keeping the industrial development away from greenfields [61]. Opposite to this statement is the finding of a Turkish study that greenfield investment is more useful for the economic growth as greenfield investments do not require additional costs, while brownfield investments need to have a direct impact on economic development [62].

Greenfields industrial park investment projects may deteriorate the soil function, which contributes to the stability of the socio-ecological system, especially the level of groundwater, production of oxygen and regulation of the climate, sources of renewable materials, plants, wood, and animals, sources of biodiversity, and human recreation with cultural and aesthetic value. Nevertheless, this fact is not included in the price of the land and the costs of the investment project. Brownfields industrial park investment projects are considered sustainable as they involve management and recovery of devastated area and return to use in a way satisfying environmental, economic, and social human needs [21]. Nevertheless, this “definition” also does not include the value of soil in the price of the land and in the costs of the investment project.

In the territory of the Slovak Republic, industrial park investment projects were supported by calls within the framework of the Sectoral Operational Program—Industry and Services. Sixteen investment projects in the brownfield and greenfield areas were supported (Table 4). Based on the data, 68% of the total supported investment projects were greenfields and only 32% were brownfields; this program period is based on the data from The Slovak Innovation and Energy Agency [63].

The industrial park investment project should be implemented at a brownfield site in the southwestern part of the eastern Slovak region, which currently belongs among the regions with the highest unemployment rate (according to the Statistical Office of the Slovak Republic) and lack of stimulus for economic and social development. In addition, the southwestern area of the region is affected by the conversion of mining and consecutive productive activities, the loss of the economic base, the increase in unemployment, large areas of devastated mining, and industrial areas of environmental concern [64]. The investment project of an industrial park with an area of 80,000 m^2^ with a building coefficient of 0.35 (built-up area is 28,322 m^2^) is characterized by a relatively high volume of investment costs of about 5.92 million Euro (Table 5). Despite this fact, the allocation of investment capital to brownfields is characterized by a high added value in the socio–economic and environmental sphere of this part of the region. It stops the devastation and decline of the area, with the construction of engineering networks and makes the conditions favorable not only for the restoration of the industrial zone but also for the entry of other entrepreneurial subjects, which directly determines the increase of the competitiveness of the industry in the region stabilizing the living standards, eliminating the negative impacts of unemployment and the environmental burden of the region threatening the environmental quality.

In the sense of the above, the decision on the allocation of investment capital should be determined by comparing selected economic indicators as shown in Table 5, which showed that the price of an 80,000 m^2^ land was 468,400 € lower for brownfield, which is mainly affected by the existing environmental burden. The total cost of new construction per m^2^ was 25 € higher on brownfield, while the cost of land preparation and construction was about 1.9 million € higher for brownfield, because this item also integrates the cost for the elimination of environmental burdens of 597,500 € as well as the costs associated with demolition and excavation works, cutting down trees and shrubs, adjusting the plan with compaction of about 333,000 €. The time needed to prepare the land for construction was 12 months longer for brownfields, which logically results from the necessity to make terrain modifications, rough terrain adjustments, and elimination of environmental burdens. The cost of constructing comparable types of objects was identical on both brownfield and greenfield; thus, the type of land in this case is insignificant, and the operational costs for the brownfield industrial park investment project were several times higher, they are increased by the cost of monitoring the environmental status of the land (about 73,000 €) or by the additional cost of building protection (about 68,000 €). The costs of environmental counselling were about 174,800 € higher for brownfield, which were related to the existing environmental burden and the demand to eliminate it, while the developer costs were quantified by 5% and were 20,200 € higher for brownfield, and the investor’s own capital requirement was about 68,000 € higher for the allocation of capital to brownfield. The return on investment was 6.6% lower for the allocation of capital to brownfield than to greenfield.

The findings of the presented study are in line with the findings of the studies of similar brownfield and greenfield areas in other countries, but some differences may be spotted due to different cultural, ethical, economic, national, governmental, and other aspects.

The results of a study using TOPSIS and SAW methods in the identification, customization, weighting, prioritization of criteria and indicators of evaluation for the determination of potential of environmental, economic, and social services in Kiasar National Park (Iran)—eight criteria and 99 indicators (environmental, economic, social and political)—were identified, while the most and least number of indicators was related to environmental and economic indicators, respectively, and the results of prioritization by TOPSIS and SAW methods revealed that environmental functions and conservation of biodiversity had a higher priority than other criteria [56]. The results of another study were obtained using Weighted Linear Combination, TOPSIS, and SAW methods in industrialization potential and industrial zoning dependence on bioenvironmental conditions in three Iranian regions. Based on the results of all the methods, the regions were suitable for industrialization, and the priority should be given to policy-making and future investment programming for industrial expansion due to specific ecological, social, and economic properties, current industrial infrastructures, and suitable population density [57].

A comparison of the economic efficiency of investing in the construction of an industrial park on a brownfield and greenfield site in USA was studied, while from the cost comparison of the implementation of the industrial park development project on brownfield and greenfields, it follows that [65]:

1. The price of the land is lower in the case of brownfield.
2. The cost of land preparation and construction is significantly higher in the case of brownfield.
3. The time necessary to prepare land for construction is significantly longer in the case of brownfield.
4. The cost of building comparable types of objects is the same, i.e., the type of land in this case is insignificant.
5. Brownfields are mainly located in the inner parts of towns and cities, where the rental as well as the cost of real estate are generally substantially lower, and the vacancy rate of the properties is higher when rented.
6. The operating costs for brownfields are higher because they are increased by the costs of monitoring the environmental status of the site and additional costs of protecting the building compared to normal costs.

From a Serbian study [9], brownfields are cost-effective for several reasons—lower costs regarding regeneration compared to new construction, lower operational costs, and investment in infrastructure. The resulting lower costs from replacing expensive energy resources has not only positive economic aspects but also environmental positives from lower fossil fuel consumption. Positive aspects, economic, environmental, and social, can also point the location of the investment to a crossroads of important roads and effective urban transport infrastructure, no negative impact on people because of the distance from the closest residential area, and protective green lines around the edges and along the roads around the investment, as well as economic development connected to new employment opportunities, with an increase in the living standard.

In India, greenfield and brownfield investment projects are employed through foreign direct investment as measures taken to solve the issues of environment for their economic and financial feasibility [13]. During the last 30 years, the shape of greenfields and brownfields has significantly changed. In 1991–2001, there was a large share of greenfield both in the urban and rural and peri-urban areas. With a growth of the urban area and its spatial expansion, it gets saturated and expands further on. This was not confirmed in the Kolkata Metropolitan Area (India) where the initial growth and compaction of the urban area gradually slowed down. In 2001–2011, greenfield development was low in the urban area, while the growth was slow in the peri-urban and rural areas. [32].

In Timisoara (Romania), brownfields were changed into spaces with one or more functionality (coworking space, maker space, community space, event space, incubator). These re-used spaces, except for several positives, were represented by their large size, low rents, and innate flexibility, and they also face problems with restrictions on the long-term use of these spaces due to financial instability of cultural operators, the public not being interested in creative activities, and pressure from the side of real-estate developers on abandoned industrial areas [33].

In re-use of brownfields as a means for the reduction of natural land and resources use, the existing decision support systems focus mainly on the economics of the project, neglecting sustainability issues. For this reason, a multi-criteria algorithm was created to determine optimal land use based on the criteria and limits according to stakeholder preferences. The criteria include aspects of sustainability and economics, including remediation costs and land value. It was applied to a case study of a former military site near Potsdam (Germany) with an emphasis on the compromise between economics and the need for sustainable development in the regional context of the brownfield site. The economic analysis showed an increase in land value due to its re-use confronted with the costs for remediation required to make the re-use. No differences in economics were found for high-cost high-value compared to low-cost low-value options. The results of the case study showed that an implementation sustainability into economics may significantly change the optimal use of abandoned land [66]. In the case study of brownfields re-use and energy transition by co-evolutionary interaction between structure and agency in rural areas of Germany, the energy transition is possible by gaining a broader agency, meaning an effective regulative, normative, and cultural–cognitive cooperation between institutions and use of plans for urban design, energy, mobility, and civic participation [67].

In Russia, industrial parks are used to develop and diversify exports, create jobs, and launch technology and knowledge sharing based on rivalry and supplier networks, the combination of geographical specificities and government policies of the industrial cluster concept. It was found that these initiatives strongly interfere with business activities and prevent competitiveness and collaboration in this area [30].

In Portugal (case study of Targus Estuary), the most significant obstacles to brownfield re-use were classified into following categories [29]:

- Governance.
- Infrastructure.
- Territorial issues.
- Finance.
- Culture.
- Environment.

In Pakistan, critical obstacles to brownfield re-use were identified by the Fuzzy Delphi Method and Structural Equation Modeling. In the first stage, 41 barriers were identified through literature review and 33 barriers were identified through expert opinion. Based on survey questionnaires addressed to stakeholders involved in the brownfield re-use, the most critical obstacles were identified to be lack of policy incentives, the complexity of public–private partnerships, lack of professional and technical personnel, the conflict between stakeholders, and lack of awareness of environmental law. The study also confirmed that critical barriers to brownfield re-use vary by country and the most critical are political and legal, financial and economic, technical and operational, management system, and environmental barriers with the most dominant being technical and operational barriers [68]. In a different study, a contextual relationship-based model using an integrated methodology of Interpretive Structural Modeling and cross-impact matrix multiplication applied to classification analyses was applied for an identification of the obstacles to brownfield re-use adoption in Pakistan. The results identified financial constraints including lack of capital and cost consideration as the most critical ones together with ownership constraints, public protests, lack of transparent communication among stakeholders, and lack of environmental justice [12].

## 6. Conclusions

Due to regional disparities in Slovakia, brownfields and greenfields are considered key for the development of society. Industrial parks are attractive for investors and may significantly improve not only the domestic market and international cooperation but also engineering, environmental, legal, and economic development of the region, including environmental impact, which, not only in the case of brownfields but also greenfields may affect the final decision on the investment. Brownfield and greenfield industrial park investment projects located in the East Slovak region were analyzed by the SAW method.

Summarizing the comparative economic indicators, in the case of the investment project of an industrial park in the southwestern part of the East Slovak region, it is preferable to allocate the investment capital to the greenfield because the return on investment was 9.5%, while in the case of brownfield, it was only 2.9%, based on the average return on the investment project expressed as a percentage of the average annual profit of the project after tax from the average value of the long-term assets acquired under the project. Nevertheless, not all the factors were in favor of greenfields, as the price of the land was lower for brownfields and the cost of constructing comparable types of objects was the same for brownfield and greenfield. Yet, the factors favoring greenfields were in prevalence, namely the following:

- The total cost of new construction.
- The cost of land preparation.
- The time needed for preparation of the land.
- The costs of environmental counselling.
- The developer costs.
- The investor’s own capital requirement.

The study has some limitations. The SAW method was used for decision-making with 11 factors that were identified on the allocation of investment projects of industrial parks implementation and the quantification of weights according to the methodological procedure with determination of their priority in the decision-making process. The selection was made based on professional publications and manuals of Slovak and foreign authors, legislative regulations, and various EU statistical databases, expert opinions, etc. Future research may consider other sources and other factors as well as use other methods for evaluation. The results were based on local situation on Slovakia; however, they may be generalized for any other region with disparities, and the used method may be adopted for any brownfields and greenfields in the world.

## Figures and Tables

**Table 1:** Types of brownfields from an economic point of view [36,37].

Brownfields	Economic Description
Suitably located	The market will “take care” of them. Possible public non-monetary intervention can increase the benefits for the local community.
Less suitably located	Public intervention and, where appropriate, the involvement of public funds, which are the cost gap of the project, is necessary. The usual ratio of public and private investment is from 1:5 or more in this case.
Non-commercial locations	Require more social goals or environmental protection. The share of public funds is higher than the private funds, usually 1:4.
Critical state	Dangerous, health- or environment-threatening. If it is no longer possible to find and bring the originator of pollution to the responsibility, the removal of damages is paid out of public funds.

**Table 2:** Quantification of decision-making factors weight.

Factor	f1	f2	f3	f4	f5	f6	f7	f8	f9	f10	f11	Σ	*α*_i_
**f1**	0	0.5	0.5	0	0	1	0.5	1	0.5	0.5	1	2.0	0.05
**f2**	0.5	0	1	0.5	0.5	1	1	1	0.5	0.5	1	7.5	0.19
**f3**	0.5	0	0	0.5	0.5	0.5	0.5	0	0.5	0.5	1	4.5	0.11
**f4**	1	0.5	0.5	0	1	0.5	1	0.5	0.5	0.5	1	7.0	0.17
**f5**	1	0.5	0.5	0	0	0.5	0.5	0	0	0	0.5	3.5	0.09
**f6**	0	0	0.5	0.5	0.5	0	1	0	0.5	0.5	0.5	1.5	0.04
**f7**	0.5	0	0.5	0	0.5	0	0	0	0.5	0	0.5	1.5	0.04
**f8**	1	0	1	0.5	1	1	1	0	0.5	1	0.5	3.5	0.09
**f9**	0.5	0.5	0.5	0.5	1	0.5	0.5	0.5	0	1	0.5	3.0	0.07
**f10**	0.5	0.5	0.5	0.5	1	0.5	1	0	0	0	1	3.0	0.07
**f11**	1	0	0	0	0.5	0.5	0.5	0.5	0.5	0	0	3.5	0.09
Sum												40.5	1.00

Note: Meaning of colors: in the matrix (f1-f11), gray—“0” was plotted on the diagonal of the matrix, yellow—“0” signals the factor was considered less important than the one it was compared to, orange—“0.5” meant the factors were of the same importance, red—“1” indicates that the factor considered was more important than the one it was compared to; those outside the matrix are only used to emphasize the results; *α*_i_—the weight of the *i*-th criterion defined by the decision-making body; Σ*α*_i_ = 1 (defined in Equation (1)).

**Table 3:** A comparison of the investment projects of brownfield and greenfield industrial parks implementation.

Factor	*α*_i_	Brownfield		Greenfield	
		Points	Σ	Points	Σ
existing transport infrastructure	0.05	4	0.198	1	0.05
existing technical infrastructure	0.19	4	0.741	1	0.19
existing manufacturing and other objects	0.11	4	0.444	1	0.11
purchase price of the land	0.17	2	0.346	4	0.69
quality connection to the regional and global freight transport system	0.09	4	0.346	2	0.17
the cost of landscaping	0.04	1	0.037	4	0.15
contact with existing subcontractors and services	0.04	3	0.111	2	0.07
environmental burden	0.09	1	0.086	4	0.35
additional costs of the investment project	0.07	1	0.074	4	0.3
operating costs	0.07	2	0.148	2	0.15
time of the plot preparation	0.09	2	0.173	4	0.35
Sum		2.70		2.57	

Note: Meaning of colors: yellow—the weight of the criterion, brown—brownfield points and sum, green—greenfield points and sum; *α*_i_—the weight of the *i*-th criterion defined by the decision-making body; Σ*α*_i_ = 1 (defined in Equation (1)).

**Table 4:** Supported investment projects of industrial parks in Slovakia [63].

**Location of Industrial Park**	**Region**	**Type of Industrial Park**
Poprad	Prešov	greenfield
Snina	Prešov	brownfield
Lipany	Prešov	greenfield
Vranov nad Topľou	Prešov	greenfield
Prešov	Prešov	greenfield
Jaklovce	Košice	brownfield
Kojšov	Košice	brownfield
Trebišov	Košice	brownfield
Hnúšťa	Banská Bystrica	brownfield
Detva	Banská Bystrica	brownfield
Lučenec	Banská Bystrica	greenfield
Vígľaš	Banská Bystrica	greenfield
Myjava	Trenčín	greenfield
Galanta	Trnava	greenfield
Hurbanovo	Nitra	greenfield
Diakovce	Nitra	greenfield

**Table 5:** The quantification of the investment costs of brownfield and greenfield industrial park implementation in the southwestern part of Eastern Slovakia.

**Factor**	**Brownfield**	**Greenfield**
Land use data		
Land area (m^2^)	80,000	80,000
Built-up area (m^2^)	28,322	0
Building coefficient (built-up area/land area)	0.35	0
The current number of landowners	2	2
Data on land development costs		
Land price (€)	719,976	1,188,342
Price of the land preparation for construction		
Removing environmental issues (€)	597,491	0
Other land preparation costs (€)	3,314,745	2,980,745
Construction costs		
Cost of construction (€)	196,176	196,176
Other costs (€)	5885	1962
Soft cost		
Legal (€)	73,027	29,211
Others—planning, designing (€)	98,918	98,918
Environmental consulting (€)	179,247	4481
Building loan cost (€)	560,977	272,190
Subtotal (€)	5,746,442	5,106,025
Developer expenses 5% (€)	172,393	152,185
Total cost (€)	5,918,835	5,258,210
Total cost per m^2^ of new construction (€)	209	184
Operating cash flow		
Number of tenants	3	3
Market rent (€)	1,057,748	1,224,656
Vacancy rate—market vacancy (%)	10	7
Object guard costs (€)	136,586	68,293
Costs to monitor the state of the environment (€)	73,027	0
Net operating income (€)	848,105	1,156,363
Financing and investing		
Loan amount (€)	5,642,131	5,274,514
Cash flow before tax (€)	8133	20,331
Net capital requirement (€)	282,107	214,080
Return on investment (%)	2.9	9.5
Length of territory preparation for construction (months)	18	6
